# Individual and institutional factors linked to high research productivity among physicians in the Arab Gulf region: implications for healthcare policy

**DOI:** 10.3389/fpubh.2026.1767901

**Published:** 2026-02-13

**Authors:** Khaldoon Al-Roomi, Amer Almarabheh, Madhawi Albuainain, Rahaf AlMuhanadi, Hessa AlSubaie, Hissa Alabbasi, Fatema Aljowder, Sara Janahi, Salman Alzayani

**Affiliations:** 1 Department of Family and Community Medicine, College of Medicine and Health Sciences, Arabian Gulf University, Manama, Bahrain; 2 Department of Internal Medicine, Military Hospital, Riffa, Bahrain; 3 Department of Obstetrics and Gynecology, King Hamad University Hospital, Al Sayh, Bahrain; 4 Department of Medical Oncology, Bahrain Oncology Center, Muharraq, Bahrain; 5 Department of Radiology, King Hamad University Hospital, Al Sayh, Bahrain; 6 Department of Pathology, King Hamad University Hospital, Al Sayh, Bahrain; 7 Department of Internal Medicine, Salmaniya Medical Complex, Manama, Bahrain

**Keywords:** Arab Gulf, Bahrain, gender disparities, healthcare policy, physicians, research productivity

## Abstract

**Introduction:**

Although physicians’ research productivity contributes to evidence-based practice and health-system learning, productivity is uneven, and little is known about individual-level traits linked with high output in Arab Gulf settings. This study examined physician characteristics associated with higher scientific publication productivity in Bahrain.

**Methods:**

Cross-sectional study of practicing physicians in Bahrain across primary and secondary healthcare and government/private sectors. Data included age, gender, years of experience, career level, sector and care setting, and lifetime number of peer-reviewed publications. High productivity was defined as ≥5 publications. Associations were examined using chi-square tests and multivariable logistic regression (SPSS v30.0).

**Results:**

In total, 239 physicians were responded to the survey (mean age 37.58 ± 11.15 years; 66.5% female; 75.3% government sector; 71.1% secondary care). One in five (≈20%) reported ≥5 publications, while about one-third had none. In univariate analyses, higher productivity was associated with male gender, age ≥40 years, ≥15 years’ experience, advanced career level, and secondary practice (all *p* ≤ 0.001). In multivariable modelling, adjusted odds ratios (ORs) were produced for employment in secondary healthcare remained strongly associated with high productivity (OR 7.364, 95% CI 1.992, 27.225; *p* = 0.003). Employment in the private sector was associated with lower odds compared with government sector (OR 0.634, 95% CI 0.259, 1.553; *p* = 0.319). Female gender showed a trend toward lower odds compared to their male counterparts (OR 0.449, 95% CI 0.197, 1.024; *p* = 0.057).

**Discussion:**

Research productivity among physicians in Bahrain is concentrated in a small, predominantly male, at advanced career stages and hospital-based doctors, suggesting structural and career-stage inequities in research engagement. An urgent need exists within the healthcare system for a more inclusive research culture that spans primary and secondary healthcare, government and private sectors, and male and female physicians alike.

## 1 Introduction

Research productivity of practicing doctors is acknowledged worldwide as one of the driving determinants for the quality of healthcare. Subsequently, health authorities aspire to integrate research initiatives into clinical practice since this would lead to better health outcomes, more efficient healthcare services, and would create a culture of excellence in health service delivery. Published research shows that healthcare organizations which encourage, and foster research have generally a more positive outcome on most of the main quality indicators, even after adjustments are made for resources and staffing differences (1, 2). Further, meta-analysis reports demonstrate that when there is a strong research culture at the healthcare workplace, it produces numerous benefits, including higher staff retention rates, improved clinical decision-making and better patient satisfaction (3, 4). Building research capacity and scientific culture among health professionals, even in the relatively well-resourced high-income countries results in significant enhancements to both, individual and group work productivity (5).

In the Gulf Cooperation Council (GCC) countries, research publications have increased significantly over the past two decades. Nevertheless, such scientific productivity remains below the expected international standards. For instance, while studies from Kingdom of Saudi Arabia present a marked increase in health-related sciences research output, further constraints for improved gains remain in view of the shortness of the protected time that health professionals have, clinical workloads, and limited healthcare organizational incentives for conducting and publishing research (6–8).

Overall, studies conducted on health professionals in the GCC countries suggest a favorable attitudinal environment toward research but with limited support facilities to conduct physician-led research in the Arab Gulf region and undertaking and publishing research does not attain the needed support. The situation in the Kingdom of Bahrain is not much different, with only one published cross-sectional study that addressed doctors’ perceptions toward research but without investigating the determinants of doctors’ scientific productivity. Similarly, findings from the Kingdom of Saudi Arabia indicate that doctors identified structural and organizational barriers as the key reasons for their limited research involvement (6, 7, 9–11).

Several knowledge gaps remain in the literature on research capacity, culture, and productivity in the GCC countries since most existing studies in this region have focused on attitudes, perceived barriers rather than on individual doctors’ scientific productivity and its socio-demographic and professional determinants (6–11). Further, there is limited published research work examining how specific doctors’ traits—such as age, gender, years of experience, clinical setting (primary vs. secondary care), employment sector (government vs. private), and career level—relate to levels of scientific productivity among practicing physicians (12–15).

The research question addressed in this paper is “which individual and institutional-level characteristics are associated with high scientific publication productivity among practicing physicians in the Kingdom of Bahrain?”. This study seeks to guide healthcare policy makers to the most effective approaches for research capacity building at the level of individuals, healthcare institutions, and the national healthcare systems. Moreover, it is hoped that the findings of this study would provide practical insights for strengthening high-quality medical research.

## 2 Methods

### 2.1 Study design and setting

A cross-sectional survey was conducted among licensed practicing physicians in the Kingdom of Bahrain working across primary and secondary healthcare and in both governmental and private healthcare sectors. The study aimed to identify individual and institutional-level characteristics associated with higher scientific publication output among physicians.

### 2.2 Sample source

The sampling frame targeted licensed physicians currently practicing in Bahrain across healthcare delivery settings. To enhance representation of physicians working in different sectors and levels of care, recruitment was implemented through broad dissemination using professional distribution channels available to the study team. The survey link directed participants to an online information sheet and consent statement prior to participation in order to reduce administrative burden on clinicians. An online self-administered questionnaire was employed seeking information on demographic data (age, gender, healthcare sector of employment).

### 2.3 Inclusion and exclusion criteria

Inclusion criteria were: (1) licensed physician, (2) currently practicing clinically in Bahrain at the time of data collection, and (3) provided informed consent and completed the questionnaire. Exclusion criteria were: (1) non-physician health professionals (e.g., nurses, pharmacists, allied health workers), and (2) medical students.

This study focused exclusively on licensed physicians because they are the professional group most consistently expected—through postgraduate training, academic promotion pathways—to lead and disseminate clinical research. The inclusion of other health professionals (e.g., nurses, pharmacists, allied health workers) would have introduced substantial heterogeneity in the collected data. Accordingly, the sampling frame and analyses were designed to identify physician-specific individual and institutional determinants of high scientific publication.

### 2.4 Data collection

Data were collected using an online self-administered questionnaire. Participation was voluntary and anonymous, and no personally identifying information was collected. The instrument captured self-reported demographic and professional characteristics and self-reported number of publications.

### 2.5 Questionnaire development and content validity

The questionnaire was developed based on the study objectives, variables commonly examined in the physician research productivity literature, and contextual relevance to Bahrain’s healthcare workforce. Questionnaire items were generated to cover demographic characteristics, employment and practice setting, professional seniority, and number of publications. Content validity was ascertained through expert review by a two of the researchers with the appropriate expertise (e.g., clinical medicine, public health/health services research, and research methods) to ensure the relevance, clarity, and comprehensiveness of the data collection tool. This process has confirmed that the questionnaire items were relevant and justifiable across the medical specialties, and that the questionnaire variables were aligned with the planned statistical analysis.

### 2.6 Pilot testing

Prior to full implementation of the study, the questionnaire was piloted with a small group of practicing physicians to assess feasibility, clarity, and completion time. This procedure has provided feedback on item wording and interpretation of the questions. Accordingly, minor modifications were implemented.

### 2.7 Study variables

The primary outcome was scientific publication productivity measured as number of publications. Publication output was categorized as none, one, two to four, and five or more articles. In addition, the number of scientific publications from each physician were ascertained. High research productivity was defined as five or more publications. High research productivity was defined as ≥5 publications (dependent variable is outcome: ≥5 vs. <5). This was based on the currently practiced institutional promotion benchmarks in the GCC countries, where 3–4 publications are usually required for progression in medical career (16). Independent variables included age group, gender, years of professional experience, career level (entry, intermediate and advanced), healthcare sector of employment (government vs. private), and clinical setting (primary vs. secondary healthcare).

### 2.8 Statistical analysis

Data were analyzed using SPSS (version 30.0). Descriptive statistics were produced as frequencies and percentages. Univariate associations between high research productivity and physician characteristics were assessed using the chi-square test. Multivariable logistic regression analysis was conducted to estimate adjusted odds ratios (OR) and 95% confidence intervals (CI) for factors independently associated with high productivity (OR did not include the value of 1.0). *p*-value less than 0.05 was considered statistically significant.

### 2.9 Ethical considerations

This study was approved by the Research and Ethics Committees of the College of Medicine and Health Sciences at Arabian Gulf University (approval number: E41-PI-9-22). The names of doctors who answered the questionnaire were kept anonymous. Confidentiality of all collected data was maintained.

## 3 Results

Table 1 presents the socio-demographic characteristics of the doctors who responded to the survey (*n* = 239). Around two-thirds of the participants in the sample were females (66.5%) and this is similar to the gender distribution pattern of the doctors in the Kingdom of Bahrain. Most of the doctors were under the age of 40 years (64.0%) with work experience less than 15 years (61.1%). The largest proportion of doctors were employed in the governmental health sector (75.3%), working mainly in secondary healthcare (71.1%) and mostly at an advanced career level (46.0%). Interestingly, only one out of five doctors has published five or more papers during their entire medical career, while on the other hand one third of the doctors had no publication record at all.

**Table 1:** Socio-demographic characteristics of doctors (*n* = 239).

Characteristic	*n* (%)
Gender	
Male	80 (33.5)
Female	159 (66.5)
Age group^*^	
<30 years	75 (31.4)
30–39 years	78 (32.6)
40–49 years	42 (17.6)
≥50 years	44 (18.4)
Years of work experience^**^	
0–4 years	55 (23.0)
5–9 years	53 (22.2)
10–14 years	38 (15.9)
15–19 years	22 (9.2)
≥20 years	71 (29.7)
Undergraduate curriculum	
Problem-based	101 (42.3)
Conventional	138 (57.7)
Clinical specialty	
Primary healthcare	69 (28.9)
Secondary healthcare	170 (71.1)
Employer	
Government	180 (75.3)
Private sector	59 (24.7)
Career level	
Entry	72 (30.2)
Intermediate	57 (23.8)
Advanced	110 (46.0)
Number of research paper publications	
None	73 (30.5)
1 article	44 (18.4)
2–4 articles	74 (31.0)
≥5 articles	48 (20.1)

* Mean ± SD = 37.58 ± 11.15.

** Mean ± SD = 13.63 ± 10.91.

The association between the doctors’ characteristics and higher publications productivity (publishing 5 research papers or more) is presented in Table 2. There were statistically significant relationships between publishing five papers or more and being a male doctor (*p* = 0.013), 40 years or older (*p* < 0.001), having a work experience of 15 years or more (*p* < 0.001), being at an advanced career level (*p* < 0.001) and employed in the secondary healthcare (*p* < 0.001). However, no significant relationship was found between the scientific productivity and the type of medical curriculum in the medical school from which the doctors have graduated (*p* = 0.964). In addition, there was no relation between the doctors’ employment sector and scientific productivity (*p* = 0.065).

**Table 2:** Traits of doctors associated with higher number of publications (*n* = 239).

Characteristic	Number of publications				*χ*^2^ value	*p*-value
	None *n* (%)	1 *n* (%)	2–4 *n* (%)	≥ 5 articles *n* (%)		
Gender						
Male	18 (22.4)	12 (15)	25 (31.3)	25 (31.3)	10.776	0.013^*^
Female	55 (34.6)	32 (20.1)	49 (30.8)	23 (14.5)		
Age group						
<30 years	43 (57.3)	15 (20)	14 (18.7)	3 (4)	95.314	<0.001^*^
30–39 years	24 (30.8)	15 (19.2)	33 (42.3)	6 (7.7)		
40–49 years	3 (7.1)	9 (21.4)	17 (40.5)	13 (31)		
≥50 years	3 (6.8)	5 (11.4)	10 (22.7)	26 (59.1)		
Years of work experience						
0–4 years	32 (58.2)	8 (14.5)	12 (21.8)	3 (5.5)	97.597	<0.001^*^
5–9 years	26 (49.1)	13 (24.5)	12 (22.6)	2 (3.8)		
10–14 years	8 (21.1)	9 (27.7)	18 (47.4)	3 (7.8)		
15–19 years	3 (13.6)	4 (18.2)	11 (50)	4 (18.2)		
≥20 years	4 (5.6)	10 (14.1)	21 (29.6)	36 (50.7)		
Undergraduate curriculum						
Problem-based	29 (28.7)	19 (18.8)	32 (31.7)	21 (20.8)	0.280	0.964
Conventional	44 (31.9)	25 (18.1)	42 (30.4)	27 (19.6)		
Clinical specialty						
Primary healthcare	24 (34.8)	24 (34.8)	18 (26.1)	3 (4.3)	27.400	<0.001^*^
Secondary healthcare	49 (28.8)	20 (11.8)	56 (32.9)	45 (26.5)		
Employer						
Government	62 (34.4)	34 (18.9)	53 (29.4)	31 (17.2)	7.238	0.065
Private sector	11 (18.6)	10 (16.9)	21 (35.6)	17 (28.9)		
Career level						
Entry	44 (61.1)	11 (15.3)	14 (19.4)	3 (4.2)	83.343	<0.001^*^
Intermediate	22 (38.6)	16 (28.1)	14 (24.6)	5 (8.8)		
Advanced	7 (6.4)	17 (15.5)	46 (41.8)	40 (36.4)		

* Statistically significant.

To identify doctors’ traits that are independently associated with a higher number of scientific research publications (publishing five research papers or more), a multivariate regression modelling analysis was conducted – Table 3. There were statistically independent significant relationships between publishing 5 papers or more and being employed in secondary healthcare (odds ratio for secondary healthcare = 7.364, CI (1.992, 27.225), *p* < 0.003). In addition, a doctor who was employed in the private sector is less likely to have published five papers or more compared with doctors employed in the government (odds ratio for private sector = 0.634, CI (0.259, 1.553), *p* = 0.319) but the difference was not statistically significant. A trend was also noted in gender (odds ratio for female gender = 0.449, CI (0.197, 1.024), *p* = 0.057), with male doctors being more likely to produce higher number of scientific publications compared to their female counterpart.

**Table 3:** Multivariable regression modelling analysis of doctors’ characteristics associated with number of publications (*n* = 239).

Characteristics	Total number	<5 articles	≥5 articles	Adjusted odds ratio		
				Odds ratio	95% C.I	*p*-value
		*n* (%)	*n* (%)			
Total	239					
Gender						
Male	80	55 (28.8)	25 (52.1)	1.00		0.057
Female	159	136 (71.2)	23 (47.9)	0.449	0.197, 1.024	
Age group						
<40 years	153	144 (75.4)	9 (18.8)	1.00		0.088
≥40 years	86	47 (24.6)	39 (81.2)	4.609	0.794, 26.736	
Years of work experience						
<15 years	146	138 (72.3)	8 (16.7)	1.00		0.339
≥15 years	93	53 (27.7)	40 (83.3)	2.458	0.389, 15.514	
Undergraduate curriculum						
Problem-based	101	80 (41.9)	21 (43.8)	1.00		0.109
Conventional	138	111 (58.1)	27 (56.3)	0.507	0.221, 1.163	
Clinical specialty						
Primary healthcare	69	66 (34.6)	3 (6.3)	1.00		0.003^*^
Secondary healthcare	170	125 (65.4)	45 (93.8)	7.364	1.992, 27.225	
Employer						
Government	180	149 (78)	31 (64.6)	1.00		0.319
Private sector	59	42 (22)	17 (35.4)	0.634	0.259, 1.553	
Career level						
Entry/intermediate	129	121 (63.4)	8 (16.7)	1.00		0.118
Advanced	110	70 (36.6)	40 (83.3)	2.459	0.797, 7.589	

* Statistically significant.

## 4 Discussion

This study provides one of the first physician-level analyses of traits associated with high scientific productivity in an Arab Gulf population. It is disappointing to find that among surveyed doctors, only one in five had published five or more research papers during their entire medical career, whereas nearly one-third of doctors had no publication record at all. Scientific productivity was higher among male doctors, older age groups (≥40 years), those with 15 or longer years of experience, physicians working in secondary healthcare, and those at advanced career levels in the univariate analyses. In the multivariable modelling, employment in secondary care and working in the government sector were associated with publishing 5 or more articles, while a trend indicated that male doctors are more likely than female doctors to achieve this high level of productivity. These findings suggest that in Bahrain, scientific publishing is concentrated among a relatively small subset of experienced, hospital-based physicians, often in advanced positions with gender and sectoral disparities in research output.

Our findings have direct implications for the quality of healthcare and the role of research in enhancing health systems. Studies from western countries show that research-active organizations tend to have better clinical outcomes in the form of lower mortality rates and better key health outcome (1, 2, 17–19). Research-engaged clinicians are more likely to adopt evidence-based therapies which would obviously improve the quality of health services (1–3, 19). Thus, it is alarming that research productivity is concentrated in a narrow subset of secondary healthcare physicians since this would unfortunately limit the extent to which these system-level benefits are enjoyed across the whole healthcare sector (primary and secondary healthcare).

The particularly low proportion of highly research productive physicians in primary care in our sample is noteworthy. Primary healthcare is critical for the prevention of diseases, management of chronic disorders, and patient-centred care. In addition, primary healthcare research is essential for generating context-relevant evidence on the whole health sector rather than only hospital-based data (9, 20–22). The under-representation of primary care physicians among the highly productive researchers in this study gives the impression that we are missing opportunities to generate locally relevant data on primary care patients, population health interventions, and continuity of care. This finding is in line with previously published work from Bahrain and Saudi Arabia demonstrating that there are several structural barriers to research in primary healthcare—such as lack of time, limited mentorship, and weak incentives—despite primary healthcare doctors exhibiting positive attitudes toward research (6, 7, 9, 11, 23–25).

Furthermore, the observed association between advanced career level and higher productivity highlights how research can reinforce professional development. Internationally, research output is a major determinant of academic promotion, leadership roles, and access to competitive funding (12, 13, 26). We believe that aligning promotion criteria and leadership selection processes with transparent and equitable recognition of research contributions could both reward existing productivity and incentivize broader engagement in research across health professionals career stages.

Our findings support prior evidence from within the GCC countries as well as other Arab populations. Studies from Saudi Arabia have shown that research productivity among faculty members varies widely, with higher outputs associated with senior academic rank, access to resources, and affiliation with research-intensive institutions (6, 7, 27, 28). The pattern observed in this study of greater scientific research productivity among older, more experienced physicians working in secondary healthcare mirrors this direction and suggests that similar structural determinants operate in Bahrain as well.

At the regional level, there is currently an uneven growth in medical research output across the Arab countries, with a concentration of publications in a small number of institutions and persistent deficiencies in research infrastructure and funding (8, 14, 27, 28). Our physician-level data from Bahrain provide micro-level corroboration of these macro-level patterns in the Arab world, showing that even within a single country with a relatively small population like Bahrain, research activity is not evenly distributed across specialties, sectors, or career stages.

The strong independent association between working in secondary healthcare and high research publication reflects that hospital-based settings often provide greater access to research data, infrastructure, collaborators, and academic networks compared with primary healthcare (3, 5, 9, 24, 25). Similarly, the higher odds ratio of publishing five or more research articles among physicians employed in the government sector compared to those in the private sector (although it was not statistically significant), may suggest differences in organizational culture, flexibility, or incentives for research between the two healthcare sectors. Another plausible explanation is that doctors employed in the government sector in Bahrain need to publish research papers to advance their clinical and academic careers.

The gender gap pattern observed in our study—a higher proportion of male doctors among those with five or more publications and a trend toward lower odds ratio of high productivity among women doctors —is also in line with the global and regional literature. A recent systematic review across academic medicine demonstrated that women faculty have consistently lower h-indices than men across specialties and ranks (12, 29, 30). Another study found that men are substantially more likely to reach full professorship at a shorter duration than women, even after adjusting for academic productivity and years in practice (13, 29, 30). This gender disparity in research publications may reflect unequal access to protected research time during working hours, lack of mentorship along with traditional family duties of female physicians in Bahrain. Given that our study has identified a similar trend in this sample of doctors from Bahrain suggests that gender inequities do exist in research opportunities which should require immediate amendments in health policy.

The generally low overall research productivity in our sample is disappointing and is consistent with earlier studies from Bahrain and Saudi Arabia that report positive attitudes but relatively modest research output among clinicians (6, 7, 9–11). Barriers for conducting research that were commonly reported in those studies—including lack of protected time, insufficient methodological training, absence of mentorship, and limited institutional incentives—are likely to underlie the patterns that were observed in our study and should be considered in future capacity-building strategies. This differential access to mentorship, disproportionate caregiving responsibilities, or biases in authorship and grant opportunities (12, 13, 15). Evaluations of targeted interventions—such as structured mentoring programs, tailored research skills workshops, and transparent promotion criteria—would provide much-needed evidence on strategies to promote gender equity in scientific productivity in the Bahraini context.

Within the process of critical thinking, one of the main findings of this study namely the low scientific productivity among physicians in general and among female doctors in particular aligns with recent published reports from other Asian countries, e.g., Pakistan (31, 32). Research has identified several factors that have contributed to such poor scientific productivity including, the limited protected time available for doctors to conduct research activities, lack of organizational or academic incentives and the unavailability of targeted mentorship programs (31, 32). From a policy perspective, gender-focused research is warranted to identify specific barriers faced by female physicians in Bahrain the study’s findings underscore the need for a coordinated national strategy to strengthen research capacity across the health system. Within this context, implementation of structured outcomes-based mentorship programs that specifically target women physicians, particularly in the primary healthcare sector is essential. In addition, providing protected research time and research methodological support is necessary.

This study has several strengths. It is, to our knowledge, the first to systematically explore the traits associated with high scientific productivity among practicing physicians in Bahrain, using individual-level data. The sample included doctors from multiple sectors (government and private), clinical settings (primary and secondary healthcare), and career stages, thereby providing a broad picture of the medical workforce. Further, the use of multivariable regression allowed the identification of independent associations between specific traits and high publication output.

On the other hand, there are some limitations to be acknowledged. The cross-sectional design cannot distinguish whether working in secondary healthcare enables greater research productivity or whether more research-oriented physicians tend to select such settings. The adjusted odds ratios for the subgroups in the multivariable analysis had wide confidence intervals. Hence, physicians’ categories were collapsed into two sub-groups to obtain better precision of the risk estimates. While the relationships between the factors linked to high research productivity among physicians were in the same direction of the univariate analysis, only employment in secondary healthcare remained statistically significant in the logistic regression model. We also note that the quantity of publications was measured rather than quality or impact (e.g., journal impact factors, citation counts, or h-index) which may be considered in future research (12–14, 26, 29, 30). However, while this study was conducted in Bahrain, there is no reason to believe that the situation would be different in other Arab countries and the findings are likely to have relevance for other healthcare systems seeking to strengthen clinician-led research (6–9, 14, 15, 27, 28).

In conclusion, this cross-sectional study of physicians’ scientific productivity in the Kingdom of Bahrain reveals that high scientific productivity is concentrated among a relatively small group of mainly male, older, more experienced, hospital-based doctors. An urgent need exists within the healthcare system for a more inclusive research culture that spans primary and secondary healthcare, government and private sectors, and male and female physicians alike. Within this context, we believe that our study offers a roadmap to address the gaps in the scientific research productivity in the Arab Gulf region.

## Data Availability

The raw data supporting the conclusions of this article will be made available by the authors, without undue reservation.
